# Machine Learning–Based Risk Prediction for Coronary Heart Disease Complicated by Hyperhomocysteinemia: Retrospective Study

**DOI:** 10.2196/80809

**Published:** 2026-03-19

**Authors:** Ming-Yuan Du, Meng-Ke Lyu, Hai-long Liu, Yi-zhuo Li, Hai-feng Yan, Xiao-hui Li

**Affiliations:** 1 Heart Center, The First Affiliated Hospital of Henan University of Chinese Medicine, National Regional (TCM) Cardiovascular Diagnosis and Treatment Center, Zhengzhou, China; 2 Collaborative Innovation Center of Prevention and Treatment of Major Diseases by Chinese and Western Medicine, Zhengzhou, China; 3 The First Affiliated Hospital of Henan University of Traditional Chinese Medicine, No. 19 Renmin Road, Jinshui District, Henan Province, Zhengzhou, 450000, China, 86 15649856289

**Keywords:** retrospective study, hyperhomocysteinemia, coronary heart disease, machine learning, predictive model

## Abstract

**Background:**

Hyperhomocysteinemia (HHcy) is recognized as an independent risk factor for coronary heart disease (CHD), yet accurately predicting CHD risk in patients with HHcy remains a challenge. This study aimed to develop and validate multiple machine learning models for predicting CHD risk in patients with HHcy and elucidate key predictors using Shapley Additive Explanation (SHAP) algorithms.

**Objective:**

This study aims to develop and validate machine learning models for predicting the risk of coronary heart disease in individuals with normal homocysteine levels, aiming to improve early risk stratification and clinical decision-making.

**Methods:**

This single-center retrospective study collected data from patients who were diagnosed with HHcy through electronic medical records, which were randomly divided into training (n=364, 70%), validation (n=78, 15%), and test (n=78, 15%) sets. Seven machine learning models were constructed, including logistic regression, k-nearest neighbor, decision tree, random forest, extreme gradient boost, light gradient boosting machine (LightGBM), and stacking. Six core variables (age, weight, hypertension, continuous drinking history, activated partial thromboplastin time, and carotid plaque) were utilized as inputs, with performance evaluation metrics encompassing area under the receiver operating characteristic curve, accuracy, *F*_1_-score, calibration curve, Brier score, and decision curve analysis. Additionally, SHAP interpretation was conducted on the optimal LightGBM model.

**Results:**

The LightGBM model exhibited superior performance in the test set (area under the receiver operating characteristic curve=0.807, *F*_1_-score=0.606), demonstrated good calibration (Brier score=0.2415), and yielded high clinical net benefit. SHAP analysis revealed age and activated partial thromboplastin time as the most influential predictors, followed by hypertension, weight, carotid plaque, and continuous drinking history. The correlation heat map illustrated low collinearity among variables, ensuring model stability.

**Conclusions:**

The LightGBM model demonstrated high accuracy and interpretability in forecasting CHD risk among patients with HHcy. The integration of machine learning and interpretable artificial intelligence methods holds promise for delivering personalized early risk assessment and intervention strategies in clinical settings.

## Introduction

Hyperhomocysteinemia (HHcy) predisposes individuals to heightened and premature susceptibility to coronary heart disease (CHD) [1,2]. Existing research has substantiated that HHcy expedites atherosclerosis progression via various mechanisms, including vascular endothelial impairment, pro-oxidative stress, and disrupted coagulation pathways, establishing it as a stand-alone CHD risk factor [3-5]. Nevertheless, the CHD risk profile in HHcy cohorts is heterogeneous, potentially modulated by a confluence of factors encompassing age, biochemical milieu, lifestyle choices, and vascular morphology.

The current assessment tools for CHD risk in patients with HHcy are limited [6]. Traditional models, such as Cox or logistic regression, are typically based on a small number of variables and struggle to capture the intricate nonlinear interactions among variables in real clinical settings. Furthermore, these models often rely on predictors that necessitate specialized knowledge or image interpretation, hindering their widespread adoption among noncardiovascular specialist clinicians [7]. Given that HHcy is a common condition and many patients are initially diagnosed in primary or nonspecialist clinics, there is a pressing need for a predictive tool that offers predictability, universality, and interpretability to facilitate early risk assessment and intervention in clinical practice.

Machine learning technology holds potential for predicting the likelihood of diabetes, arrhythmia, and various cardiovascular diseases by effectively capturing intricate multidimensional patterns [8]. Ensemble models, such as light gradient boosting machine (LightGBM) and extreme gradient boosting (XGBoost), excel in automatically discerning key features and enhancing predictive accuracy [9]. Nevertheless, there is a scarcity of dedicated modeling research on CHD risk among individuals with HHcy. Moreover, the clinical interpretability of numerous machine learning models remains inadequate, constraining their reliability and utility in medical settings [10,11].

This study seeks to develop CHD risk prediction models for individuals with HHcy using real-world electronic health record data from a single center [12]. The study aims to compare the performance of 7 mainstream machine learning algorithms in terms of discriminative power, calibration, and clinical net benefit. To improve clinical interpretability, the Shapley Additive Explanation (SHAP) method was used to assess model decision-making and identify key variables. Emphasis was placed on the generalizability of variable selection and form, prioritizing standardized biochemical and lifestyle indicators that are routinely accessible to avoid reliance on specific specialties or complex imaging techniques. The objective is to create a practical model that can be easily implemented across various clinical settings, balancing predictability and interpretability. This model aims to serve as an early risk assessment tool for high-risk HHcy populations and establish a basis for future multicenter external validation studies.

## Methods

### Research Materials

**Figure 1.**
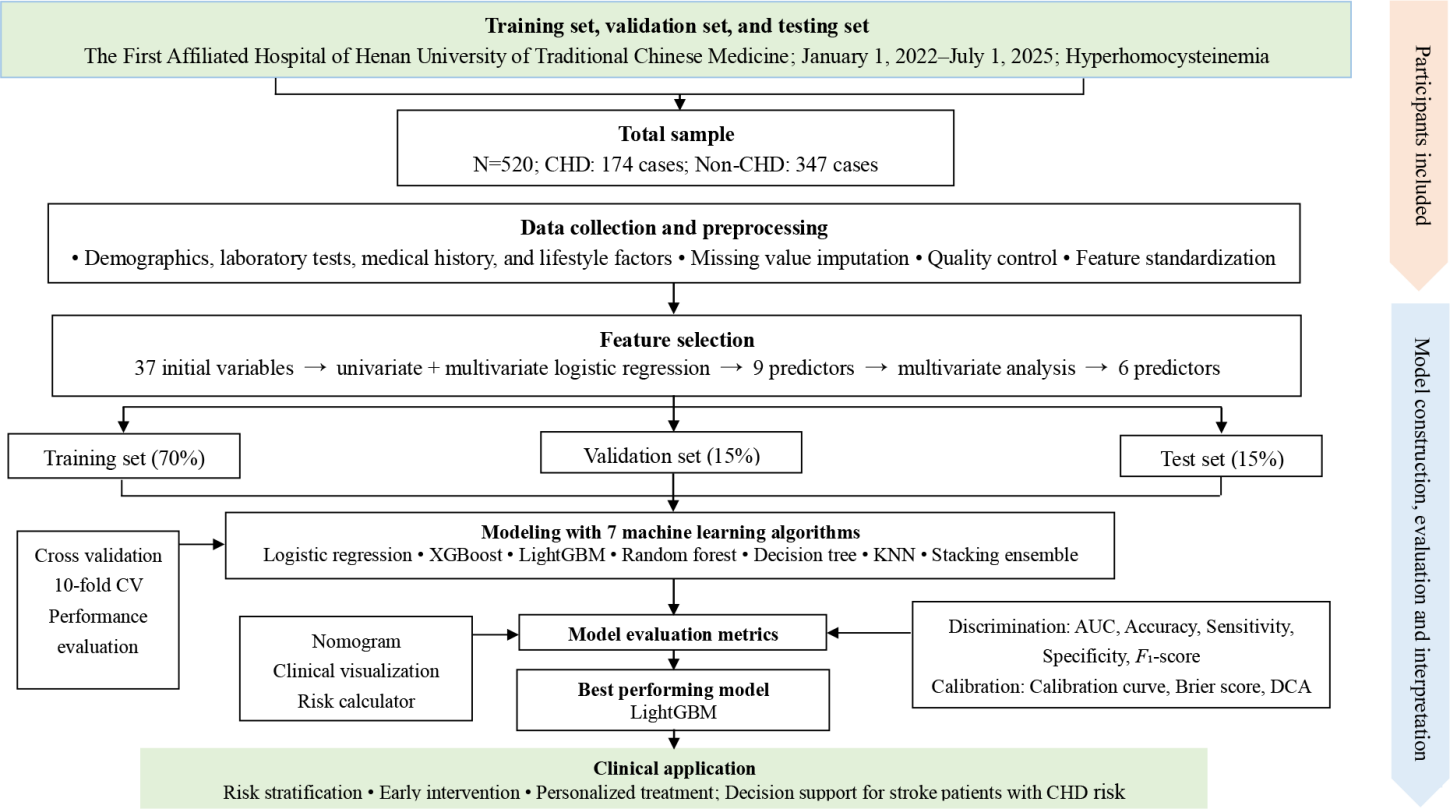
Flowchart of developing and validating machine learning–based models for predicting coronary heart disease (CHD) risk in patients with hyperhomocysteinemia. This flowchart outlines the entire process of constructing and evaluating risk prediction models for CHD in patients with hyperhomocysteinemia. AUC: area under the receiver operating characteristic curve; CV: cross-validation, DCA: decision curve analysis; KNN: k-nearest neighbor; LightGBM: light gradient boosting machine; XGBoost: extreme gradient boosting.

A comprehensive workflow of the study, including data collection, feature selection, model development, evaluation, and interpretation, is illustrated in Figure 1.

### General Information

This retrospective registry study examines patients with HHcy treated at the First Affiliated Hospital of Henan University of Traditional Chinese Medicine between January 1, 2022, and July 1, 2025.

Patients were categorized into either the CHD group or the non-CHD group based on clinical diagnoses. Subsequent to admission, demographic features, laboratory values, medical history, familial history, and additional indicators were gathered for statistical assessment.

### Ethical Considerations

Ethical approval was obtained from the Ethics Committee of the First Affiliated Hospital of Henan University of Chinese Medicine (approval number: 2025HL-202‐01). The data used were fully deidentified, and no personally identifiable information was accessible to the researchers. The requirement for informed consent was waived by the ethics committee in accordance with national regulations, citing the retrospective nature of the study. No compensation was provided to participants.

### Diagnostic Criteria

The patient was diagnosed with stable CHD in accordance with the 2025 American College of Cardiology/American Heart Association (ACC/AHA) guideline for chronic coronary syndromes [13]. Acute coronary syndromes were identified following the criteria outlined in the 2023 ESC and 2021 ACC/AHA guidelines for non–ST-elevation acute coronary syndrome [14,15].

### Inclusion and Exclusion Criteria

#### Inclusion Criteria

Participants were required to meet the diagnostic criteria for HHcy, be aged between 18 and 80 years, and have complete baseline clinical data available for analysis.

#### Exclusion Criteria

Participants excluded from the study encompass those with comorbid severe organic conditions (eg, advanced tumors, hepatorenal insufficiency), nonatherosclerotic heart ailments (eg, congenital heart disease, myocarditis), individuals with mental illness or cognitive deficits hindering study cooperation, pregnant or lactating women, and those engaged in other clinical trials within the preceding 3 months.

### Observation Indicators

This study utilized data collected through a standardized case report form, encompassing five key categories of core indicators: (1) demographics and basic information, such as age, gender, height, weight, BMI, educational level, and marital status; (2) medical history and lifestyle factors, including a history of diseases, such as hypertension, diabetes, transient ischemic attack, as well as lifestyle habits, such as persistent smoking and alcohol consumption; (3) laboratory examination indicators, comprising blood routine parameters (red blood cell, white blood cell, and platelet counts), biochemical markers (total cholesterol, triglycerides, and creatinine), and coagulation function tests (activated partial thromboplastin time [APTT], prothrombin time, and fibrinogen); (4) imaging examination indicators, specifically the presence or absence of carotid plaques as determined by ultrasound or computed tomography angiography; and (5) quality control measures involving the uniform extraction of all data by trained researchers through the electronic medical record system and laboratory information system. A double-person review system was implemented to ensure data accuracy. Following the integrity assessment, no missing values were identified in the variables included, thus obviating the need for imputation or data elimination procedures. The detailed data extraction workflow and quality control procedures are provided in Multimedia Appendix 1.

### Machine Learning Modeling and Related Statistical Methods

Statistical analysis was performed using SPSS Statistics 27. Machine learning modeling utilized R 4.5 (caret package) and Python 3.13 (scikit-learn, xgboost, lightgbm) libraries. Quantitative data were reported as mean (SD). Group differences were assessed using independent-sample 2-tailed *t* tests (for normal distribution) or Mann-Whitney *U* tests (for nonnormal distribution), based on data distribution. Categorical data were presented as n (%), with group differences analyzed using the chi-square test or Fisher exact probability method.

### Variable Screening and Dataset Division

Variable screening involved a 2-step dimensionality reduction approach utilizing “univariate+multivariate logistic regression.” Initially, 9 variables exhibiting significant differences (*P*<.05) between the CHD and non-CHD groups were selected for the candidate pool. Subsequently, multivariate analysis was conducted using stepwise regression (backward method, inclusion criterion *α*=.05, exclusion criterion *α*=.10), resulting in the retention of 6 independent predictors: age, body weight, hypertension, history of continuous alcohol consumption, APTT, and carotid artery plaque. The variance inflation factors for all variables were less than 5, indicating the absence of significant multicollinearity.

The data were stratified based on the presence of CHD comorbidity, resulting in division into a training set (n=364), a validation set (n=78), and a test set (n=78) at a ratio of 7:1.5:1.5. To ensure result reproducibility, a random seed (random_state=2024) was set. The training set was used for model training and initial parameter optimization, the validation set for fine-tuning model parameters and classification threshold optimization, and the test set as an independent dataset for the final model performance assessment.

### Machine Learning Model Construction

Seven classification models were developed to forecast the risk of CHD, categorized into traditional models (logistic regression, decision tree, and k-nearest neighbors [KNNs]) and ensemble models (random forest, XGBoost, LightGBM, and stacking). Hyperparameter optimization was performed on all models using grid search (GridSearchCV) on the training set to enhance the area under the receiver operating characteristic curve (AUC) on the validation set.

Logistic regression employs L2 regularization and class weight balancing (class_weight='balanced') with a regularization strength of C=0.1. The decision tree and random forest models optimize the max_depth, which is ultimately determined to be 7, with min_samples_split set to 2 for the decision tree and n_estimators set to 50 for the random forest. KNN utilizes the Euclidean distance metric, with the number of neighbors ultimately set to 9.

In the context of boosting models, both XGBoost and LightGBM were configured with a scale_pos_weight of 2 to address class imbalance. Parameter optimization yielded the following settings: XGBoost (max_depth=3 and learning_rate=0.3) and LightGBM (max_depth=3 and learning_rate=0.01). The stacking model used the aforementioned 6 models as base learners, with logistic regression serving as the meta-learner. The integration of outputs was achieved through 5-fold cross-validation.

All parameter tuning details are listed in Table S2 in Multimedia Appendix 2 to enhance reproducibility.

### Model Evaluation and Validation

A multidimensional index is used to thoroughly assess the model’s performance, with the subsequent metrics computed on the training, validation, and test sets, respectively:

Discrimination is primarily assessed using the AUC, computed through “sklearn.metrics.roc_auc_score.” Additionally, metrics, such as accuracy, recall, specificity, and *F*_1_-score, are documented. To provide a comprehensive evaluation of classification performance, the precision-recall (PR) curve is generated, and the PR-AUC is determined.

Calibration is assessed through a calibration curve generated using the sklearn.calibration.calibration_curve function. An optimal calibration curve closely aligns with the diagonal line. The Brier score quantifies the accuracy of predicted probabilities by measuring the mean squared error between predicted probabilities and true labels. A lower Brier score signifies more precise predicted probabilities.

We used a 10-fold cross-validation method on the training set to assess model stability, recording the mean AUC and standard deviation. To evaluate generalization capability, we considered the metrics of the test set as the reference standard. A significant decrease in the test set’s AUC compared to the training set (eg, ΔAUC<0.1) suggested potential overfitting.

### Sample Imbalance Processing and Threshold Optimization

To address the 1:2 sample imbalance between the CHD and non-CHD groups, the following strategies were implemented to enhance the model’s ability to identify the minority class (CHD group):

For models supporting weight parameters (eg, logistic regression, XGBoost, LightGBM, and random forest), class_weight='balanced' or scale_pos_weight=2 was set. Specifically, class_weight='balanced' automatically calculates class weights inversely proportional to class frequencies, amplifying the contribution of CHD samples in the loss function. Meanwhile, scale_pos_weight=2 directly sets the weight of positive samples (CHD group) to twice that of negative samples (non-CHD group), aligning with the 1:2 sample ratio and mitigating the model’s bias toward the majority class caused by imbalance.

Although class weight adjustment balances class importance during training, the default classification threshold (0.5) may not meet the clinical demand for high sensitivity in CHD screening. Therefore, further threshold optimization was performed on the best-performing LightGBM model. Based on predicted probabilities from the validation set, candidate thresholds ranging from 0.1 to 0.6 (stepping at 0.05) were evaluated, with sensitivity, specificity, and *F*_1_-score calculated for each threshold. The optimal threshold was determined by balancing these metrics—prioritizing high sensitivity (to identify as many true CHD patients as possible) while maintaining acceptable specificity. This approach breaks the limitation of a fixed threshold, enabling the model to better align with the practical goal of “reducing missed diagnoses” in imbalanced scenarios (see Figure S1 in Multimedia Appendix 3).

### Analysis of Model Interpretability

The LightGBM model’s optimal performance was assessed using the SHAP algorithm for both global and local interpretability analyses. Globally, the SHAP summary plot illustrated the average contribution of each variable to the model prediction, identifying key factors influencing the risk of CHD. Locally, the SHAP dependence plot depicted the functional relationship between each feature and the model output, highlighting the positive and negative effects of variables, such as age and APTT, on the predicted value. Additionally, a Pearson correlation heatmap was generated to assess collinearity among features, confirming that no strong correlations (*r*<0.5) existed among the model variables. This analysis ensured the model’s stability and bolstered the credibility of its interpretation.

### Sample Size Estimation

Based on the recommendations by van Smeden et al [16,17] and Riley et al [18,19], we estimated the minimum sample size required to develop a reliable multivariable prediction model using 5 candidate predictors. Assuming an outcome prevalence of approximately 30%, a shrinkage factor of 0.9, and an expected Cox-Snell *R*² of 0.15, the minimum required sample size was calculated to be approximately 218. This study developed models with a larger sample size, thus mitigating the potential for major overestimation bias based on these parameters.

### External Benchmark Models

For comparative purposes, Framingham risk score [20] and the ACC/AHA pooled cohort equations (ASCVD score) [21] were calculated for all patients using baseline clinical parameters. These scores were included as benchmark models to evaluate the incremental value of the model.

## Results

### Comparison of Clinical Data Between the 2 Groups

This study comprised 520 patients with HHcy, with 174 in the CHD group and 347 in the non-CHD group, resulting in a CHD detection rate of 33.46%. The analysis of baseline data revealed significant differences in 9 variables between the 2 groups, all with *P*<.05, as detailed in Table 1.

**Table 1.** Comparison of baseline characteristics between patients with hyperhomocysteinemia with and without coronary heart disease (CHD)^a^.

Characteristic		Control group (n=347)	Case group (n=174)	*P* value
Demographic and general information				
Age (y), mean (SD)		65.30 (10.36)	70.49 (8.76)	<.001^b^
Sex, n (%)				<.001^b^
Male		263 (0.71)	107 (0.29)	
Female		83 (0.55)	67 (0.45)	
Height (cm), mean (SD)		167.89 (7.29)	167.62 (7.30)	.78
Weight (kg), mean (SD)		66.96 (10.60)	71.52 (9.91)	.04^b^
BMI, mean (SD)		24.73 (3.22)	25.12 (3.06)	.07
Education level, n (%)				.86
Junior high school or below		224 (0.66)	114 (0.34)	
High school and above		122 (0.67)	60 (0.33)	
Household annual income (RMB 10,000 yuan^c^), n (%)				.13
<8		280 (0.67)	150 (0.33)	
≤8		66 (0.73)	24 (0.27)	
Occupation in the 6 months prior to onset, n (%)				.66
Mental workers		43 (0.64)	24 (0.36)	
Physical workers		303 (0.74)	105 (0.26)	
Payment type, n (%)				.12
Basic medical care		232 (0.69)	102 (0.31)	
Urban medical care		113 (0.61)	72 (0.39)	
Marital status, n (%)				.65
Married		333 (0.67)	166 (0.33)	
Separated		13 (0.62)	8 (0.32)	
Body temperature		36.46 (0.29)	36.45 (0.22)	.85
Respiratory rate		18.95 (4.67)	18.68 (1.76)	.82
Heart rate		77.14 (12.62)	77.41 (12.64)	.88
Systolic blood pressure		143.87 (21.69)	141.49 (21.09)	.16
Diastolic blood pressure		84.19 (13.92)	81.67 (14.37)	.05
Medical history, n (%)				
Hypertension		209 (0.61)	134 (0.39)	<.001^b^
Diabetes mellitus		176 (0.70)	76 (0.30)	.14
Hyperlipidemia		79 (0.70)	36 (0.30)	.58
History of TIA^d^		20 (0.56)	16 (0.44)	.15
Continuous smoking history		135 (0.75)	44 (0.25)	<.001^b^
Continuous drinking history		82 (0.78)	20 (0.22)	<.001^b^
Carotid plaque		275 (0.64)	154 (0.36)	.01^b^
Blood biochemical indicators, mean (SD)				
Red blood cell count		4.46 (0.48)	4.43 (0.52)	.32
White blood cell count		6.90 (4.28)	6.61 (1.93)	.80
Platelet count		207.82 (60.41)	211.49 (53.30)	.32
Hemoglobin concentration		141.76 (29.06)	143.21 (38.77)	.15
Total cholesterol		4.42 (1.27)	4.39 (1.24)	.63
Triglycerides		1.73 (1.59)	1.65 (1.10)	.39
Low-density lipoprotein		2.63 (0.87)	2.53 (0.98)	.11
High-density lipoprotein		1.14 (0.29)	1.20 (0.30)	.06
Prothrombin time		11.37 (2.76)	10.86 (2.96)	.84
Fibrinogen content		3.15 (0.95)	3.19 (0.75)	.11
Activated partial thromboplastin time		30.52 (4.95)	29.44 (3.82)	.01^b^
Thrombin time		18.67 (64.39)	15.23 (1.89)	.34
Glycated hemoglobin		6.50 (1.63)	6.44 (1.28)	.17
Creatinine		74.44 (70.07)	65.23 (21.57)	.04^b^
Uric acid		313.56 (92.76)	310.13 (90.29)	.64

a The table includes demographic information, clinical history, lifestyle factors, and laboratory indicators for a total of 520 patients. Quantitative variables are presented as mean (SD), while categorical variables are shown as counts and percentages. Group differences were assessed using independent-sample *t* tests for normally distributed data, Mann-Whitney *U* tests for nonnormally distributed data, and chi-square tests for categorical data.

b P<.05.

c Conversion based on an exchange rate of 1 US $≈7 RMB.

d TIA: transient ischemic attack.

### Univariate Regression Analysis of Risk Factors for CHD in HHcy Patients

Nine statistically significant factors were selected as independent variables for logistic regression analysis to predict the occurrence of CHD. The results revealed that 6 variables—age, weight, hypertension, continuous drinking history, APTT, and carotid plaque—significantly influenced the occurrence of CHD (all *P*<.05), as shown in Table 2.

**Table 2.** Logistic regression analysis of risk factors for coronary heart disease (CHD) in patients with hyperhomocysteinemia.^a^

Factor	OR^b^	SE	Wald statistics	*P* value	95% CI	
Age	1.06	0.01	22.68	<.001	1.08-1.03	
Gender	0.69	0.26	2.12	.15	1.14-0.42	
Weight	1.04	0.01	13.90	<.001	1.06-1.02	
Hypertension	1.99	0.23	9.26	<.001	3.09-1.28	
Continuous smoking history	0.90	0.27	0.14	.71	1.54-0.53	
Continuous drinking history	0.51	0.33	4.03	.045	0.99-0.27	
APTT^c^	0.95	0.02	5.82	.02	0.99-0.90	
Cr^d^	0.99	0.01	1.96	.16	1.00-0.98	
Carotid plaque	1.79	0.29	3.99	.046	3.18-1.01	

a The table presents the outcomes of univariate logistic regression analysis that pinpoint factors linked to the incidence of coronary heart disease in patients with hyperhomocysteinemia. It encompasses odds ratios, SE, Wald statistics, and 95% CI for each factor.

b OR: odds ratio.

c APTT: activated partial thromboplastin time.

d Cr: creatinine.

### Establishment and Evaluation of 7 Machine Learning Models for CHD Risk Prediction in Patients With HHcy

This study utilized 7 machine learning algorithms, namely logistic regression, XGBoost, LightGBM, random forest, decision tree, KNNs, and stacking ensemble, to develop risk prediction models for CHD in patients with HHcy based on 6 core predictors: age, weight, hypertension, continuous drinking history, APTT, and carotid plaque. In the training set assessment, each model exhibited distinct performance characteristics. Notably, the decision tree, random forest, and XGBoost models achieved perfect discrimination (AUC=1.000) and classification metrics (accuracy, sensitivity, specificity, and *F*_1_-score=1.000), indicating potential overfitting, unlike the logistic regression and KNN models. The LightGBM model displayed strong performance with an AUC of 0.987, accuracy of 0.854, and *F*_1_-score of 0.818. Conversely, the stacking ensemble model yielded an AUC of 0.933, accuracy of 0.810, and *F*_1_-score of 0.660, demonstrating inferior performance compared to the aforementioned potentially overfitting models.

During the validation phase, model performance varied notably among different algorithms. The XGBoost model exhibited the highest AUC of 0.802 and an *F*_1_-score of 0.689. The stacking model outperformed others with an AUC of 0.800, the highest accuracy of 0.769, and an *F*_1_-score of 0.625. The LightGBM model achieved an AUC of 0.780 and an *F*_1_-score of 0.648. In comparison, the logistic model yielded an AUC of 0.755 and an F1-score of 0.639. The random forest model attained an AUC of 0.777; however, it demonstrated low sensitivity at 0.355 and an *F*_1_-score of 0.489. The decision tree model, with an AUC of 0.679 and an *F*_1_-score of 0.613, displayed limited generalization ability and was notably impacted by overfitting during training.

During testing, the LightGBM model demonstrated the highest AUC of 0.807, a sensitivity of 0.913, and an *F*_1_-score of 0.636, indicating promising performance. The logistic model yielded an AUC of 0.796, an *F*_1_-score of 0.603, a sensitivity of 0.826, and a specificity of 0.618, striking a balance between prediction accuracy and clinical relevance. In contrast, the stacking model achieved an AUC of 0.802 but exhibited lower sensitivity at 0.478 and an *F*_1_-score of 0.564, suggesting limited generalizability. Although the random forest model achieved the highest accuracy at 0.769, its *F*_1_-score was 0.571. The XGBoost model’s performance notably decreased, with an AUC of 0.757 and an *F*_1_-score of 0.523 (Table 3).

**Table 3.** Performance metrics of the 7 machine learning models for predicting coronary heart disease (CHD) risk in patients with hyperhomocysteinemia across different datasets.

Model and stage	AUC^a^	Accuracy	Sensitivity	Specificity	*F*_1_-score
Training					
Decision tree	1.000	1.000	1.000	1.000	1.000
KNN^b^	0.795	0.734	0.475	0.861	0.540
LightGBM^c^	0.987	0.854	0.992	0.787	0.818
Logistic	0.701	0.646	0.683	0.627	0.560
Random forest	1.000	1.000	1.000	1.000	1.000
Stacking	0.933	0.810	0.558	0.934	0.660
XGBoost^d^	1.000	1.000	1.000	1.000	1.000
Validation					
Decision tree	0.679	0.692	0.613	0.745	0.613
KNN	0.751	0.731	0.452	0.915	0.571
LightGBM	0.780	0.679	0.742	0.638	0.648
Logistic	0.755	0.667	0.742	0.617	0.639
Random forest	0.777	0.705	0.355	0.936	0.489
Stacking	0.800	0.769	0.484	0.957	0.625
XGBoost	0.802	0.756	0.677	0.809	0.689
Testing					
Decision Tree	0.656	0.615	0.696	0.582	0.516
KNN	0.768	0.744	0.565	0.818	0.565
LightGBM	0.807	0.692	0.913	0.600	0.636
Logistic	0.796	0.679	0.826	0.618	0.603
Random Forest	0.765	0.769	0.522	0.873	0.571
Stacking	0.802	0.782	0.478	0.909	0.564
XGBoost	0.757	0.603	0.739	0.545	0.523

a AUC: area under the receiver operating characteristic curve.

b KNN: k-nearest neighbors.

c LightGBM: light gradient boosting machine.

d XGBoost: extreme gradient boosting.

In conclusion, both the LightGBM and logistic regression models demonstrated consistent performance across the validation and testing phases, indicating their heightened clinical utility.

The performance of the 7 machine learning models (decision tree, KNN, LightGBM, logistic regression, random forest, stacking, and XGBoost) in predicting CHD in patients with hyperhomocysteinemia was evaluated. Performance metrics, such as area under the receiver operating characteristic (ROC) curve (AUC), accuracy, sensitivity, specificity, and *F*_1_-score, were assessed on the training, validation, and test sets. The results indicate consistent discriminative ability and stability across different data partitions, with LightGBM demonstrating superior performance in the test set (AUC=0.807, *F*_1_-score=0.636).

### Model Performance Evaluation

**Figure 2.**
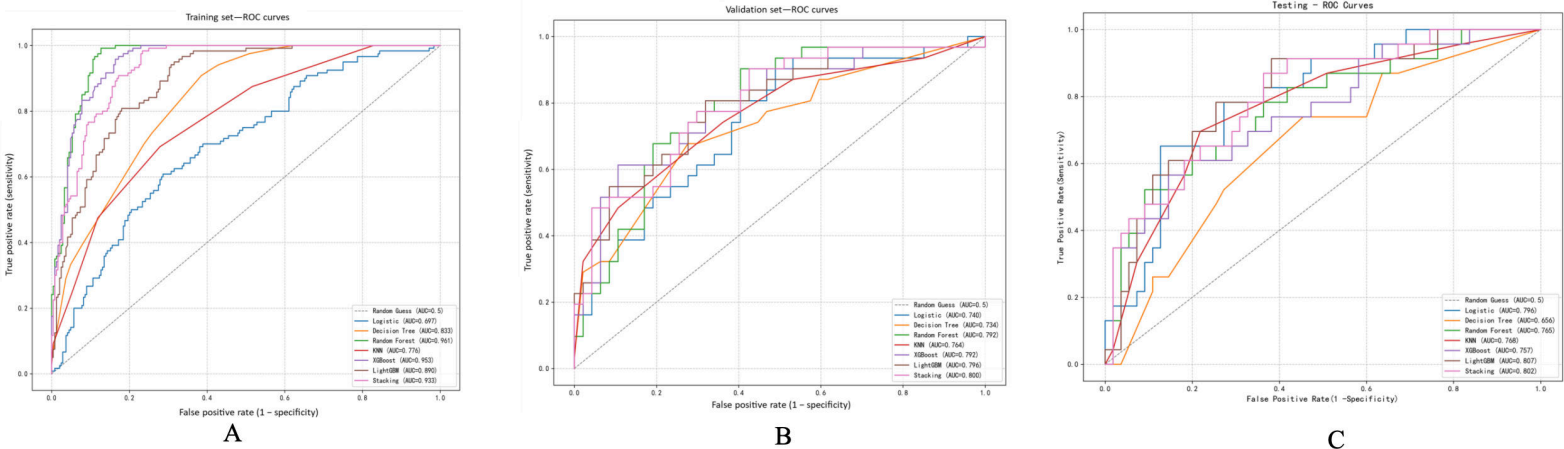
Receiver operating characteristic (ROC) curves of coronary heart disease (CHD) risk prediction models constructed by 7 machine learning algorithms in Hyperhomocysteinemia patients ROC) curves were utilized to assess the discriminative performance of 7 distinct machine learning models, including logistic regression, k-nearest neighbors (KNN), decision tree, random forest, extreme gradient boosting (XGBoost), light gradient boosting machine (LightGBM), and stacking, on various datasets: the training set, validation set, and test set denoted as (A), (B), and (C) respectively. The area under the receiver operating characteristic curve (AUC) was employed as a metric to quantify the models’ performance, where increased values are indicative of enhanced capability in discriminating between individuals with CHD and those without.

Upon analyzing the ROC curves, it was evident that within the training set, the random forest (AUC=0.961), XGBoost (AUC=0.953), stacking (AUC=0.933), and LightGBM (AUC=0.890) models displayed exceptional discriminatory capabilities, indicating potential overfitting. Conversely, the logistic regression model exhibited a comparatively lower AUC of 0.697, while the KNN and decision tree models achieved AUC values of 0.776 and 0.833, respectively (Figure 2A). In the validation set, the stacking model maintained superior performance (AUC=0.800), closely followed by LightGBM (AUC=0.796), XGBoost (AUC=0.792), and random forest (AUC=0.792). The logistic model also demonstrated satisfactory performance (AUC=0.740), surpassing the decision tree (AUC=0.734) and KNN (AUC=0.764) models (Figure 2B). Within the testing set, the LightGBM (AUC=0.807) and stacking (AUC=0.802) models achieved the highest AUC values, trailed by KNN (AUC=0.768), random forest (AUC=0.765), and XGBoost (AUC=0.757). The logistic regression model also displayed robust discriminatory ability (AUC=0.796), while the decision tree model exhibited the lowest AUC (0.656; Figure 2C).

**Figure 3.**
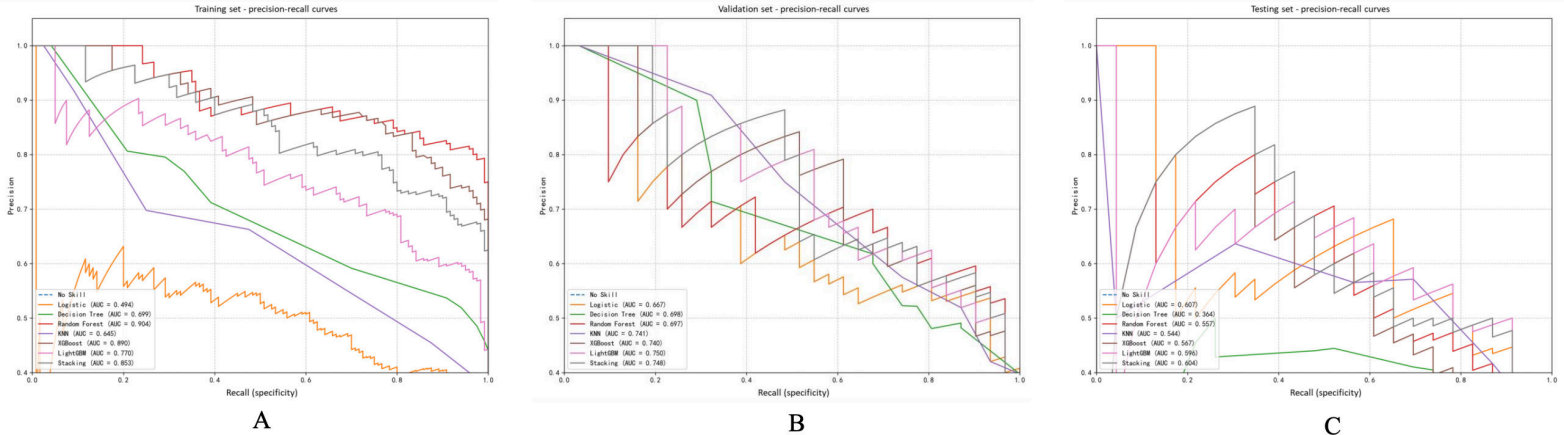
Precision-recall (PR) curves of coronary heart disease (CHD) risk prediction models constructed by the 7 machine learning algorithms. PR curves evaluate model performance in imbalanced datasets, emphasizing precision (positive predictive value) and recall (sensitivity). The results for the training set are displayed in (A), the validation set in (B), and the test set in (C). The PR-AUC (area under the precision-recall curve) is reported for each model, with greater values denoting better classification performance for the positive class (CHD). KNN: k-nearest neighbor; LightGBM: light gradient boosting machine; XGBoost: extreme gradient boosting.

Upon analyzing the PR curves, distinct variations in model efficacy were evident across the three datasets. In the training dataset, the random forest model exhibited the highest PR-AUC value of 0.904, outperforming XGBoost (0.890), stacking (0.853), and LightGBM (0.770), underscoring its robust classification capability on the internal data. Conversely, the Logistic model displayed limited discriminatory power with an AUC of 0.494, while the decision tree and KNN models achieved PR-AUCs of 0.699 and 0.645, respectively (Figure 3A).

In the validation dataset, the LightGBM model demonstrated the highest PR-AUC of 0.750, followed closely by stacking (0.748) and KNN (0.741). The XGBoost and random forest models performed comparably with AUCs of 0.740 and 0.697, respectively, whereas the logistic model yielded a PR-AUC of 0.667, and the decision tree model slightly lagged behind with an AUC of 0.698 (Figure 3B).

In the testing dataset, the LightGBM (AUC=0.596) and stacking (AUC=0.604) models exhibited moderate PR performance. The logistic model followed with an AUC of 0.607. Conversely, XGBoost (0.567), random forest (0.557), and KNN (0.544) demonstrated slightly lower PR-AUCs, with the decision tree model displaying the weakest performance (AUC=0.364; Figure 3C).

### ROC Curve Analysis of Risk Prediction Models Based on 10-Fold Cross-Validation

**Figure 4.**
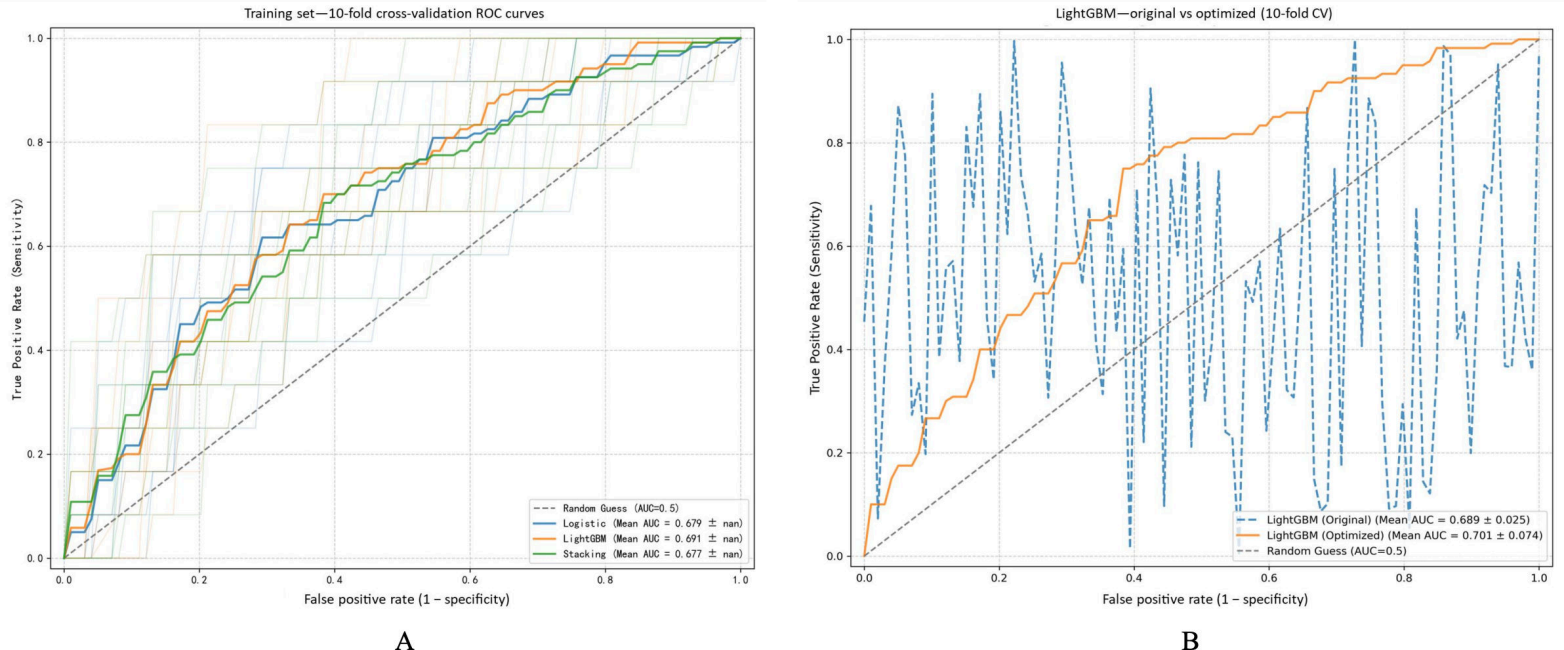
Receiver operating characteristic (ROC) curve analysis of risk prediction models based on 10-fold cross-validation. (A) ROC curves were generated for the 3 best-performing models, namely logistic regression, light gradient boosting machine (LightGBM), and stacking, through 10-fold cross-validation on the training dataset. LightGBM demonstrated the highest average area under the receiver operating characteristic curve (AUC) of 0.691, with logistic regression and stacking following closely at 0.679 and 0.677, respectively. (B) A comparison of LightGBM performance prehyperparameter and posthyperparameter optimization revealed enhancements. The optimized model exhibited a higher mean AUC of 0.701 (SD 0.074), surpassing the original model’s performance of 0.689 (SD 0.025), thus validating its increased stability.

ROC analysis using 10-fold cross-validation was used to assess the efficacy of 3 leading models: logistic regression, LightGBM, and stacking. As depicted in Figure 4A, during the training phase, the LightGBM model demonstrated the highest average AUC of 0.691, marginally surpassing logistic regression (AUC=0.679) and stacking (AUC=0.677), underscoring its superior discriminatory capability. Additionally, as illustrated in Figure 4B, following hyperparameter optimization, LightGBM’s performance was further bolstered, with its mean AUC escalating from 0.689 (0.025) to 0.701 (0.074). This enhancement substantiates LightGBM’s resilience across iterations. Consequently, LightGBM was ultimately designated as the preferred model for subsequent analyses.

### Calibration Curves and Brier Score Analysis

**Figure 5.**
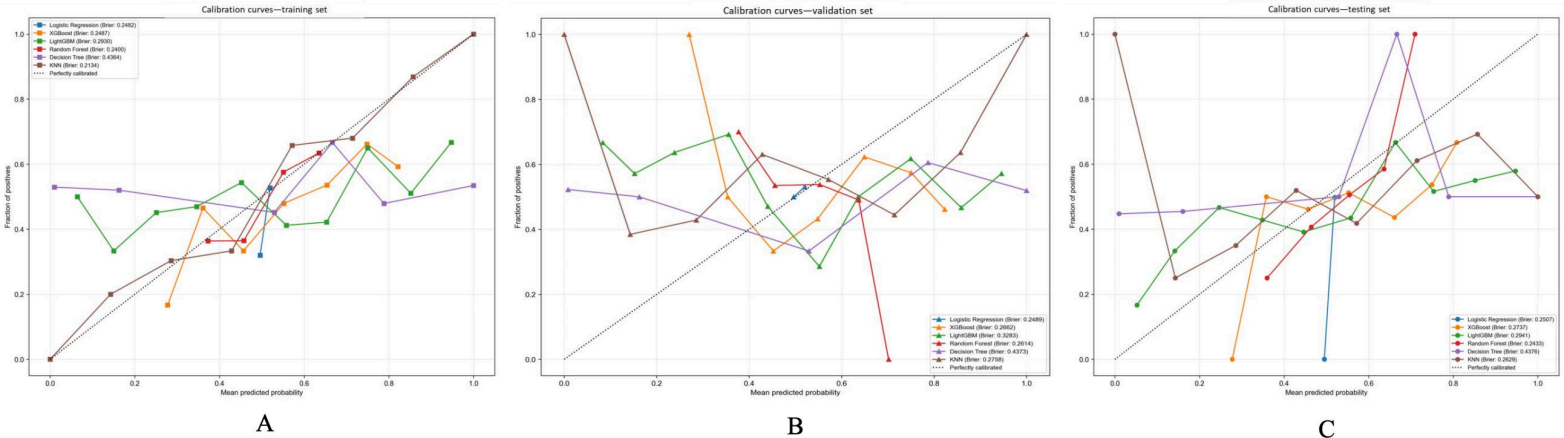
Calibration curves and Brier scores of 6 machine learning models. Calibration curves were utilized to evaluate the concordance between predicted probabilities and observed outcomes of coronary heart disease (CHD) across 3 sets: the training set, validation set, and test set. The diagonal dotted line in the graphs symbolizes ideal calibration, with Brier scores (inset) serving as a measure of prediction accuracy (lower scores indicating improved calibration). Both extreme gradient boosting (XGBoost) and k-nearest neighbors (KNN) models demonstrated consistent calibration performance across all datasets.

Calibration curves and Brier scores were utilized to assess the alignment between predicted probabilities and observed outcomes across various datasets. In the training set (Figure 5A), all models exhibited moderate calibration performance. The KNN model displayed the lowest Brier score (0.2134), closely trailed by random forest (0.240), logistic regression (0.2482), and XGBoost (0.2487). Within the validation set (Figure 5B), random forest exhibited favorable calibration (Brier score=0.2614), with XGBoost (0.2662) and KNN (0.2758) also demonstrating relatively strong performance. In the test set (Figure 5C), logistic regression demonstrated the most accurate calibration (Brier score=0.2507), followed by XGBoost (0.2737) and KNN (0.2629). While the Brier scores indicated acceptable calibration for multiple models, no single model consistently outperformed others across all 3 datasets. Nevertheless, XGBoost and KNN showcased consistent and favorable calibration performance.

### Clinical Utility Evaluation Based on Decision Curve Analysis

**Figure 6.**
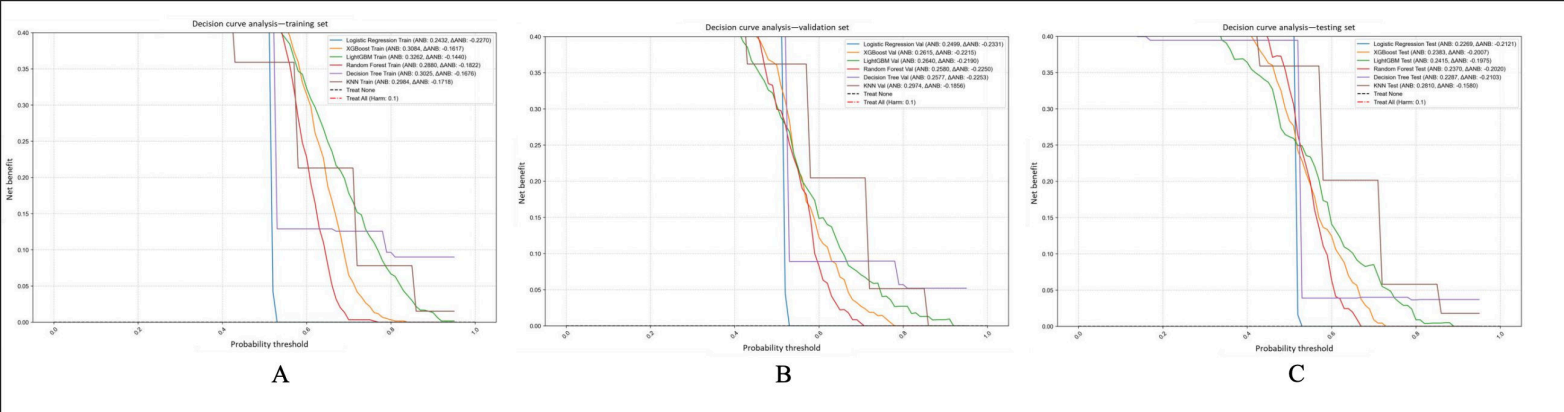
Decision curve analysis (DCA) of 6 machine learning models. The clinical utility of models is assessed by DCA through the calculation of net benefit at different probability thresholds in the training, validation, and test sets. Net benefit is determined as the difference between the proportion of correctly identified cases of coronary heart disease (CHD) and the proportion of unnecessary interventions. K-nearest neighbors (KNN), light gradient boosting machine (LightGBM), and extreme gradient boosting (XGBoost) consistently exhibited superior net benefit compared to strategies of treating all or treating none, especially within the 0.3‐0.7 threshold range.

Decision curve analysis was used to assess the clinical efficacy of various machine learning models on the training, validation, and test sets. Figure 6 illustrates that the KNN model yielded the highest average net benefit across all 3 datasets (training: 0.2984, validation: 0.2974, and test: 0.281), closely trailed by LightGBM (training: 0.3262, validation: 0.264, and test: 0.2415) and XGBoost (train: 0.3084, validation: 0.2615, and test: 0.2383). These models consistently outperformed the “Treat None” and “Treat All” strategies in terms of net benefit over a broad range of probability thresholds. Notably, LightGBM and KNN consistently demonstrated superior clinical utility, particularly within the 0.3‐0.7 threshold range, suggesting their potential for guiding personalized preventive interventions in practical healthcare settings.

### SHAP-Based Interpretation of the Optimal LightGBM Model

**Figure 7.**
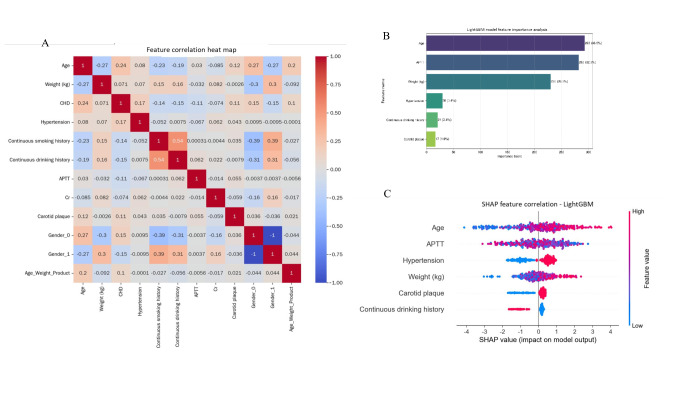
Shapley Additive Explanation (SHAP)–based interpretation and feature correlation analysis of the light gradient boosting machine (LightGBM) model. (A) A SHAP summary plot displays the average absolute impact of individual features on the prediction of coronary heart disease (CHD) risk, highlighting age and activated partial thromboplastin time (APTT) as the most influential factors. (B) A SHAP dependence plot illustrates the direction (positive or negative) and strength of feature effects on the model’s output, indicating that higher age and APTT levels are associated with an increased risk of CHD. (C) A Pearson correlation heatmap of key features demonstrates minimal multicollinearity (|*r*|<0.5 for all features except continuous drinking or smoking history, where *r*=0.54), thereby ensuring the stability of the model. Cr: creatinine.

To enhance the interpretability of the LightGBM model, SHAP values were utilized to assess the significance of individual features on the model’s predictions. The feature importance summary plot, derived from the mean absolute SHAP values, identified age, APTT, hypertension, weight, carotid plaque, and continuous drinking history as the primary predictors influencing the risk of CHD in patients with HHcy (Figure 7A). Notably, age and APTT exhibited the most substantial average impact, underscoring their pivotal roles in the model’s predictive capabilities.

The SHAP dependence plot (Figure 7B) illustrates the impact of each feature on the model output in terms of both direction and magnitude. The elevated values of age and APTT were consistently linked to a higher predicted risk of CHD, with hypertension and carotid plaque presence also notably increasing the SHAP value. Notably, the influence of continuous drinking history exhibited variability across individual patient profiles, suggesting potential interactions with other variables.

A feature correlation heatmap (Figure 7C) was generated to evaluate multicollinearity and the interplay among essential features. The heatmap reveals a moderate positive correlation between continuous drinking history and continuous smoking history (*r*=0.54) and a slight negative correlation between age and weight (*r*=–0.27). These findings endorse the incorporation of these variables into the LightGBM model without introducing unnecessary redundancy.

These results demonstrate that the LightGBM model offers both superior predictive accuracy and the ability to reveal the significance and interplay of clinical features. This capability can enhance personalized risk evaluation and targeted preventive measures for coronary heart disease in individuals with HHcy.

## Discussion

This research conducted a comparative analysis of 7 machine learning algorithms to forecast the likelihood of CHD in patients with HHcy using authentic single-center electronic health records. The models were developed utilizing 6 fundamental variables (age, weight, hypertension, history of sustained alcohol consumption, APTT, and carotid artery plaque), and their discriminatory capacity, calibration, and clinical applicability were thoroughly assessed across training, validation, and test datasets. The findings revealed that the LightGBM model exhibited superior overall performance, demonstrating the highest AUC (0.807) and a relatively elevated *F*_1_-score (0.636) in the test set, underscoring its promising clinical predictive capability.

A significant discovery is that the model does not depend on variables specific to CHD or HHcy. Instead, it opts for indicators that are commonly available and extensively standardized in routine clinical settings, including coagulation function (activated partial thromboplastin time), arterial structure markers (presence of carotid artery plaques), basal metabolism (body weight), and behavioral aspects (alcohol consumption history). These variables do not necessitate expert image analysis or sophisticated molecular assays, thereby augmenting the model’s practicality in primary health care facilities or noncardiovascular disciplines.

Upon applying the SHAP algorithm to interpret the model, we validated that age and APTT are the most impactful variables within the model. Notably, the predictive significance of APTT warrants special consideration. Serving as a marker for the intrinsic coagulation pathway, it could potentially signify a hypercoagulable state within the HHcy population. This pathological characteristic has been identified as a key factor contributing to accelerated atherosclerosis [22]. The SHAP dependence plot and correlation heatmap provided additional confirmation of the minimal collinearity among variables, thereby augmenting the interpretive clarity and reliability of the model.

The stacking model exhibits a high AUC of 0.800 in the validation set; however, its *F*_1_-score decreases to 0.33 in the test set, with a sensitivity of only 0.26, indicating limited generalization ability and a predisposition toward overfitting. Conversely, the LightGBM model demonstrates consistent performance across the training, validation, and test sets, underscoring its robustness in diverse sample scenarios. While the logistic regression model shows a slightly lower AUC, its balanced *F*_1_-score, sensitivity, and specificity underscore its reliability in terms of generalization stability.

In this study, we aim to streamline variable processing by avoiding artificial binning, segmentation, high-dimensional mapping transformations, and the creation of interaction terms. This approach is intended to enhance the model’s generalizability across different datasets by minimizing reliance on specific data structures. Additionally, we refrain from using oversampling methods, such as synthetic minority over-sampling technique, to mitigate issues related to feature distribution drift resulting from synthetic sample generation. Instead, we tackle data imbalance by adjusting category weights and optimizing thresholds.

However, this study is subject to several limitations. First, the data were sourced from a singular medical institution, potentially introducing regional, health care–seeking motivation, or detection biases. For instance, individuals with high homocysteine levels who consented to coagulation function tests may exhibit distinct health awareness and disease profiles compared to those who declined the tests. Second, the retrospective design of the study resulted in the exclusion of crucial variables that could impact CHD risk, such as dietary patterns, nutritional status, and renal function. Consequently, the interpretation of changes in serum markers remains inconclusive. Furthermore, the study solely predicted the present CHD status of patients documented in electronic health records. The absence of longitudinal follow-up data precluded the modeling of event timing or disease progression. To address these limitations, future research should integrate prospective cohorts and long-term follow-up data to validate the model’s reliability and establish a dynamic risk assessment framework.

In conclusion, the LightGBM model developed in this study exhibits exceptional predictive accuracy and interpretability in individuals with HHcy, offering a robust basis for real-world implementation. We propose its consideration as a potential tool for early CHD risk stratification to aid clinicians in promptly identifying high-risk patients. Subsequent investigations may concentrate on external validation and model refinement in a multicenter context.

## Supplementary material

10.2196/80809 Multimedia Appendix 1 Detailed data extraction and quality-control protocols.

10.2196/80809 Multimedia Appendix 2 Grid search ranges and final hyperparameters for all machine learning models.

10.2196/80809 Multimedia Appendix 3 Threshold optimization curve for the light gradient boosting machine (LightGBM) model.

## Abbreviations

ACC/AHA: American College of Cardiology/American Heart Association
APTT: activated partial thromboplastin time
AUC: area under the receiver operating characteristic curve
CHD: coronary heart disease
HHcy: hyperhomocysteinemia
KNN: k-nearest neighbor
LightGBM: light gradient boosting machine
PR: precision-recall
ROC: receiver operating characteristic
SHAP: Shapley Additive Explanation
XGBoost: extreme gradient boosting

## References

[1] Guéant JL, Guéant-Rodriguez RM, Oussalah A, Zuily S, Rosenberg I (2023). Hyperhomocysteinemia in cardiovascular diseases: revisiting observational studies and clinical trials. Thromb Haemost.

[2] Tinelli C, Di Pino A, Ficulle E, Marcelli S, Feligioni M (2019). Hyperhomocysteinemia as a risk factor and potential nutraceutical target for certain pathologies. Front Nutr.

[3] Balint B, Jepchumba VK, Guéant JL, Guéant-Rodriguez RM (2020). Mechanisms of homocysteine-induced damage to the endothelial, medial and adventitial layers of the arterial wall. Biochimie.

[4] Herrero-Fernandez B, Gomez-Bris R, Somovilla-Crespo B, Gonzalez-Granado JM (2019). Immunobiology of atherosclerosis: a complex net of interactions. Int J Mol Sci.

[5] Cetin E, Raby AC (2025). Understanding atherosclerotic plaque cellular composition: recent advances driven by single cell omics. Cells.

[6] Alaa AM, Bolton T, Di Angelantonio E, Rudd JHF, van der Schaar M (2019). Cardiovascular disease risk prediction using automated machine learning: a prospective study of 423,604 UK Biobank participants. PLoS One.

[7] Wysocki A, Fułek M, Macek P (2024). Ultrasound carotid plaque score and severity of coronary artery disease assessed by computed tomography angiography in patients with arterial hypertension. Diagnostics (Basel).

[8] Pal P, Singh HV, Grover V, Manikandan R, Karimi R, Khishe M (2025). Interactive cardiovascular disease prediction system using learning techniques: Insights from extensive experiments. Results Control Optim.

[9] Hajihosseinlou M, Maghsoudi A, Ghezelbash R (2023). A novel scheme for mapping of MVT‑type Pb–Zn prospectivity: LightGBM, a highly efficient gradient boosting decision tree machine learning algorithm. Nat Resour Res.

[10] Gulum MA, Trombley CM, Ozen M, Esen E, Aksamoglu M, Kantardzic M (2024). Why are explainable AI methods for prostate lesion detection rated poorly by radiologists?. Appl Sci (Basel).

[11] Obeagu EI, Ezeanya CU, Ogenyi FC, Ifu DD (2025). Big data analytics and machine learning in hematology: transformative insights, applications and challenges. Medicine (Baltimore).

[12] Weng SF, Reps J, Kai J, Garibaldi JM, Qureshi N (2017). Can machine-learning improve cardiovascular risk prediction using routine clinical data?. PLoS One.

[13] Williams MS, Levine GN, Kalra D (2025). Correction to: 2025 AHA/ACC clinical performance and quality measures for patients with chronic coronary disease: a report of the American College of Cardiology/American Heart Association joint committee on performance measures. Circ Cardiovasc Qual Outcomes.

[4] Byrne RA, Rossello X, Coughlan JJ (2023). 2023 ESC Guidelines for the management of acute coronary syndromes. Eur Heart J.

[15] Visseren FLJ, Mach F, Smulders YM (2022). 2021 ESC Guidelines on cardiovascular disease prevention in clinical practice. Eur J Prev Cardiol.

[16] van Smeden M, de Groot JAH, Moons KGM (2016). No rationale for 1 variable per 10 events criterion for binary logistic regression analysis. BMC Med Res Methodol.

[17] van Smeden M, Moons KG, de Groot JA (2019). Sample size for binary logistic prediction models: beyond events per variable criteria. Stat Methods Med Res.

[18] Riley RD, Snell KI, Ensor J (2019). Minimum sample size for developing a multivariable prediction model: part II—binary and time-to-event outcomes. Stat Med.

[19] Riley RD, Snell KIE, Ensor J (2019). Minimum sample size for developing a multivariable prediction model: part I—continuous outcomes. Stat Med.

[20] D’Agostino RB, Vasan RS, Pencina MJ (2008). General cardiovascular risk profile for use in primary care: the Framingham Heart study. Circulation.

[21] Goff DC, Lloyd-Jones DM, Bennett G (2014). 2013 ACC/AHA guideline on the assessment of cardiovascular risk: a report of the American College of Cardiology/American Heart Association Task Force on Practice Guidelines. Circulation.

[22] Yin H, Cheng X, Liang Y (2021). High perceived stress may shorten activated partial thromboplastin time and lead to worse clinical outcomes in patients with coronary heart disease. Front Cardiovasc Med.

