# Supplementary material for: Machine Learning–Based Risk Prediction for Coronary Heart Disease Complicated by Hyperhomocysteinemia: Retrospective Study
Source: JMIR Med Inform. 2026 Mar 19;14:e80809. doi: 10.2196/80809 (PMC13002003; doi:10.2196/80809)
Supplement: Multimedia Appendix 1 [file medinform-v14-e80809-s001.docx]

| **Factor** | **OR** | **SE** | **Wald** | **P** | **95%CI** |
| --- | --- | --- | --- | --- | --- |
| Age | 1.06 | 0.01 | 22.68 | < 0.001 | 1.03–1.08 |
| Gender | 0.69 | 0.26 | 2.12 | 0.15 | 0.42–1.14 |
| Weight | 1.04 | 0.01 | 13.90 | < 0.001 | 1.02–1.06 |
| Hypertension | 1.99 | 0.23 | 9.26 | < 0.001 | 1.28–3.09 |
| Continuous smoking history | 0.90 | 0.27 | 0.14 | 0.71 | 0.53–1.54 |
| Continuous drinking history | 0.51 | 0.33 | 4.03 | 0.05 | 0.27–0.99 |
| APTT | 0.95 | 0.02 | 5.82 | 0.02 | 0.90–0.99 |
| Cr | 0.99 | 0.01 | 1.96 | 0.16 | 0.98–1.00 |
| Carotid plaque | 1.79 | 0.29 | 3.99 | 0.05 | 1.01–3.18 |
