# Supplementary material for: Machine Learning–Based Risk Prediction for Coronary Heart Disease Complicated by Hyperhomocysteinemia: Retrospective Study
Source: JMIR Med Inform. 2026 Mar 19;14:e80809. doi: 10.2196/80809 (PMC13002003; doi:10.2196/80809)
Supplement: Multimedia Appendix 2 [file medinform-v14-e80809-s002.docx]

**Multimedia Appendix 1: Grid Search Ranges and Final Hyperparameters for All Machine Learning Models**

| **Model** | **Parameter** | **Search Range** | **Final Value** |
| --- | --- | --- | --- |
| Logistic Regression | C | [0.01, 0.1, 1, 10] | 0.1 |
| Decision Tree | max_depth | [3, 5, 7, 9] | 7 |
| Random Forest | n_estimators | [10, 50, 100, 200] | 50 |
| KNN | n_neighbors | [3, 5, 7, 9, 11] | 9 |
| XGBoost | learning_rate | [0.01, 0.1, 0.3] | 0.3 |
| LightGBM | learning_rate | [0.01, 0.1, 0.3] | 0.01 |

| **Model** | **C** | **max_depth** | **min_samples_split** | **n_estimators** | **learning_rate** | **n_neighbors** |
| --- | --- | --- | --- | --- | --- | --- |
| Logistic | 0.1 |  |  |  |  |  |
| Decision Tree |  | 7 | 2 |  |  |  |
| Random Forest |  | 7 |  | 50 |  |  |
| KNN |  |  |  |  |  | 9 |
| XGBoost |  | 3 |  |  |  | 0.3 |
| LightGBM |  | 3 |  |  |  | 0.01 |
