# Supplementary figures and images for: Machine Learning–Based Risk Prediction for Coronary Heart Disease Complicated by Hyperhomocysteinemia: Retrospective Study
Source: JMIR Med Inform. 2026 Mar 19;14:e80809. doi: 10.2196/80809 (PMC13002003; doi:10.2196/80809)

Changes in sensitivity and specificity at different thresholds

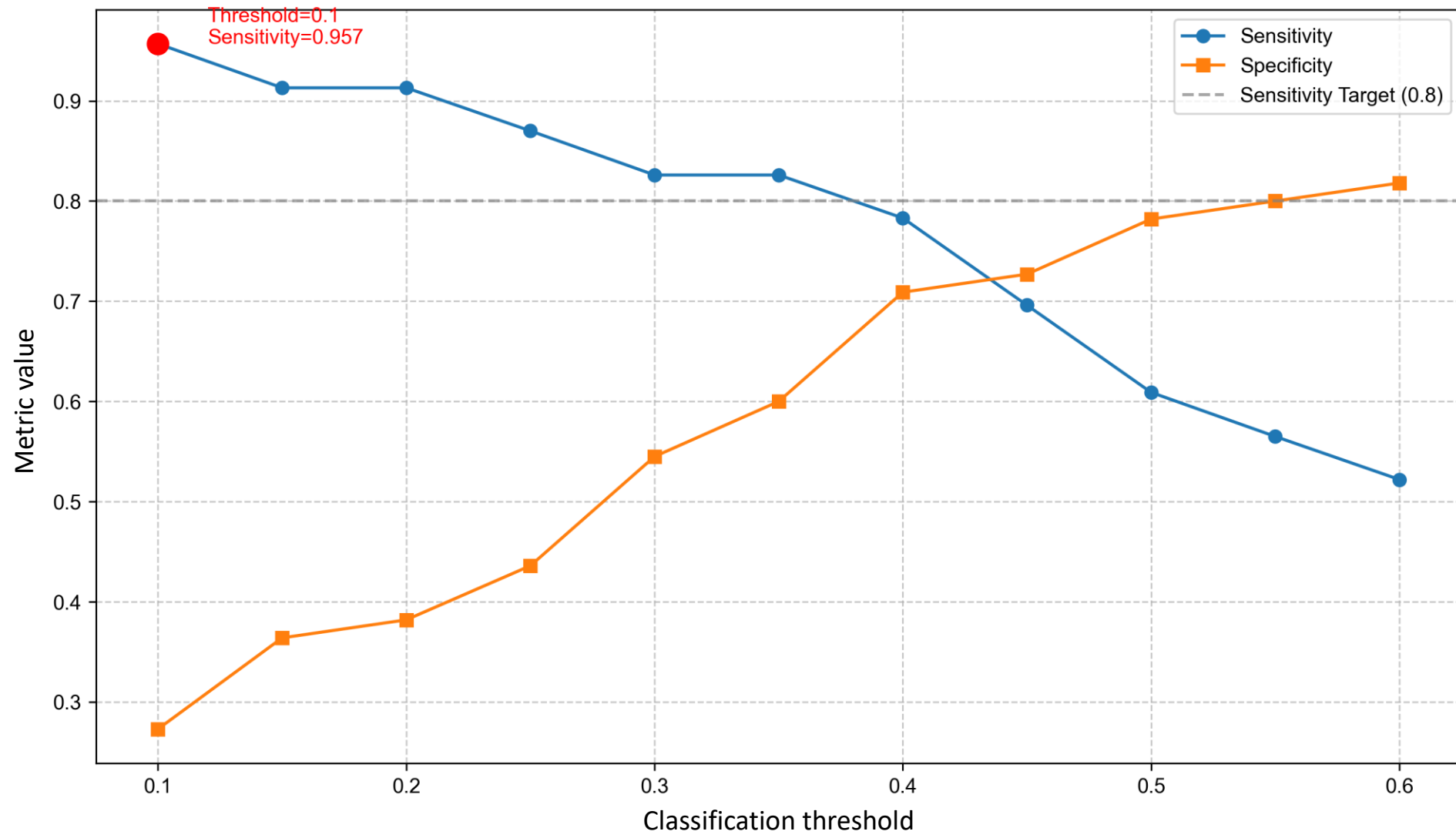

Supplement: Multimedia Appendix 3 [file medinform-v14-e80809-s003.pdf]
